# Plant growth-promoting rhizobacteria enhance metal tolerance in rice (*Oryza sativa* L.) under chromium and lead stress

**DOI:** 10.1038/s41598-026-61223-0

**Published:** 2026-07-19

**Authors:** Rana M. Alshegaihi, Muna Abdul-Rahman Al-Malki, Dalia Mohammad Melebari, Hanan El Sayed Osman, Dikhnah Alshehri, Suliman Mohammed Suliman Alghanem, Khalid Ali Khan, Amany H. A. Abeed, Sharifullah Sharifi

**Affiliations:** 1 Department of Biological Sciences, College of Science, University of Jeddah, 21493 Jeddah, Saudi Arabia; 2 Department of Biology, College of Science, Umm Al-Qura University, Makkah, Saudi Arabia; 3 Botany and Microbiology Department, Faculty of Science, Al-Azhar University, Cairo, Egypt; 4 Department of Biology, Faculty of Science, University of Tabuk, 71491 Tabuk, Saudi Arabia; 5 Department of Biology, College of Science, Qassim University, 52571 Burydah, Saudi Arabia; 6 Center of Bee Research and its Products (CBRP); and Biology Department, College of Science, King Khalid University, P.O. Box 9004, 61413 Abha, Saudi Arabia; 7 Botany and Microbiology Department, Faculty of Science, Assiut University, Assiut, 71516 Egypt; 8 Department of Science and Irrigation, Faculty of Plant Sciences, National Agricultural Sciences and Technology University, Kandahar, Afghanistan

**Keywords:** Heavy metal stress, Redox regulation, Cereal crop resilience, Phytoremediation strategies, Nutrient uptake efficiency, Biotechnology, Environmental sciences, Microbiology, Plant sciences

## Abstract

In the current industrial scenario, chromium (Cr) and lead (Pb) contamination poses a serious threat to agricultural ecosystems due to their toxic effects on plant growth and productivity. However, limited studies have explored the role of plant growth-promoting rhizobacteria (PGPR) in alleviating combined Cr and Pb stress in rice. Therefore, the present study investigated the potential of *Pseudomonas putida*, *Bacillus amyloliquefaciens*, and *Pseudomonas fluorescens* in mitigating metal-induced toxicity in rice (*Oryza sativa* L.) under different Cr and Pb levels [0 (without Cr and Pb stress), 100 and 200 mg kg⁻^1^)]. The research outcomes indicated that elevated levels of Cr and Pb stress in the soil significantly (*P* ≤ 0.05) decreased plant growth, biomass, photosynthetic pigments, and gas exchange characteristics, while increasing oxidative stress biomarkers including malondialdehyde (MDA) and hydrogen peroxide (H₂O₂). Metal toxicity also altered antioxidant defense systems, sugar accumulation, proline metabolism, and the ascorbate–glutathione (AsA–GSH) cycle. However, the application of *P. putida*, *B. amyloliquefaciens*, and *P. fluorescens* significantly improved morphophysiological and biochemical attributes, enhanced antioxidant defense and related gene expression, reduced oxidative damage, and decreased Cr and Pb accumulation in plant tissues. Furthermore, PGPR application improved cellular fractionation and regulated stress-responsive metabolic pathways in *O. sativa* under metal-stressed conditions. Overall, the findings highlight the significant potential of these PGPR strains as sustainable biological agents for improving metal stress tolerance and physiological performance in *O. sativa* plants grown under Cr and Pb toxicity.

## Introduction

Environmental stresses are of major concern in agricultural production due to their adverse effects on crop growth, yield, plant physiological performance, and soil ecological health. Among these stresses, heavy metal accumulation in soils has emerged as a serious threat because of its phytotoxic effects and negative impact on soil organisms and overall agricultural sustainability^1–4^. Heavy metals include cadmium (Cd), lead (Pb), nickel (Ni), cobalt (Co), iron (Fe), zinc (Zn), chromium (Cr), iron (Fe), arsenic (As), silver (Ag) and the platinum group elements^5^. Contamination of agricultural soils with Cr and Pb has become one of the most toxic and widespread environmental problems^4^. Photosynthesis, respiration, cell division, water relations, opening and closing of stomata, nitrogen metabolism, and mineral nutrition are the main metabolic processes within the plants, which are negatively affected by Cr and Pb stress^6,7^. Although Cr is toxic for plant growth, it is easily taken by the roots and then transported to the shoots where it can cause retorted growth, stunted root development, reduce branching, alteration in photosynthesis and respiration, diminished nutrient uptake, blocked electron transport chain as well as changed the membrane permeability^8^. Higher Pb levels in plants cause ultrastructural alterations, oxidative stress in plants, and increased electrolyte leakage (EL) and malondialdehyde (MDA) concentrations, whereas induced alterations in antioxidant enzyme activities such as superoxide dismutase (SOD), peroxidase (POD), and catalase (CAT) and ascorbate peroxidase (APX)^9^. Hence, it is immensely required to safeguard plant from Cr and Pb toxicity to counter the phytotoxicity and oxidative stress triggered by the uptake of Cr and Pb in plants.

Plant growth-promoting rhizo-bacteria (PGPR) help in improving plant growth and metal resistance^10^, by modifying the concentration of growth regulators and phytohormones that facilitate the plant’s ability to tolerate metal contaminants^11^. PGPR improve Cr and Pb bioremediation because of their ability to enhance heavy metal bioavailability, uptake, and conversion into less toxic forms through methylation, oxidation, demethylation, and reduction^11^. *Pseudomonas putida* is a well-known PGPR with significant capabilities in promoting plant growth. This bacterium has been found to enhance plant resilience by modulating physiological and biochemical processes, particularly under stressed environments^12^. Similarly, *Pseudomonas fluorescens* is recognized for its biofilm formation, siderophore production, and biocontrol properties, which collectively contribute to improved plant health and resilience under environmental stressors^13,14^. Moreover, *Bacillus amyloliquefaciens* has been extensively studied for its ability to improve plant physiological performance, chlorophyll synthesis, and rhizospheric activity^15^. The application of these PGPR under stressed conditions has shown promising results in under stressed conditions has shown promising results in boosting plant growth, increasing nutrient absorption, and reducing oxidative stress^14,16^. Therefore, these PGPR demonstrates significant potential as a beneficial agent in sustainable agriculture, presenting a promising strategy to enhance crop resilience against a range of abiotic stresses. Rice (*Oryza sativa* L.) is a cereal grain, it is the most widely consumed staple food for a large part of the world’s human population, especially in Asia and Africa. *O. sativa* cultivation is well-suited to countries and regions with low labor costs and high rainfall, as it is labor-intensive to cultivate and requires ample water^17^.

Globalization has increased the accumulation of Cr and Pb in the environment, posing a serious threat to the growth and productivity of *O. sativa*. Considering the agricultural importance of *O. sativa* as a major cereal crop, the present study was designed to investigate the potential role of different PGPR in improving the growth and physiological performance of *O. sativa* under Cr and Pb-contaminated soil conditions. Although the beneficial effects of PGPR under metal-stressed environments have been widely reported, comparative information regarding the efficiency of different PGPR strains in regulating antioxidant defense systems, cellular integrity, proline metabolism, metal detoxification, and stress-responsive biochemical pathways in *O. sativa* under combined Cr and Pb stress remains limited. Furthermore, the mechanistic responses associated with different PGPR strains under simultaneous Cr and Pb toxicity are still insufficiently understood. Therefore, this study hypothesized that different PGPR strains exhibit distinct abilities to alleviate Cr and Pb-induced phytotoxicity through modulation of physiological, biochemical, antioxidant, and cellular defense mechanisms in *O. sativa*. To address this knowledge gap, the present study evaluated the effects of different PGPR strains on (I) morphological traits and photosynthetic efficiency, (II) oxidative stress biomarkers together with enzymatic and non-enzymatic antioxidant responses and their associated gene expression, and (III) proline metabolism, AsA–GSH cycle, cellular fractionation, and Cr and Pb uptake in different plant parts under metal stress conditions.

## Materials and methods

### Experimental setup and treatments

**Fig. 1:**
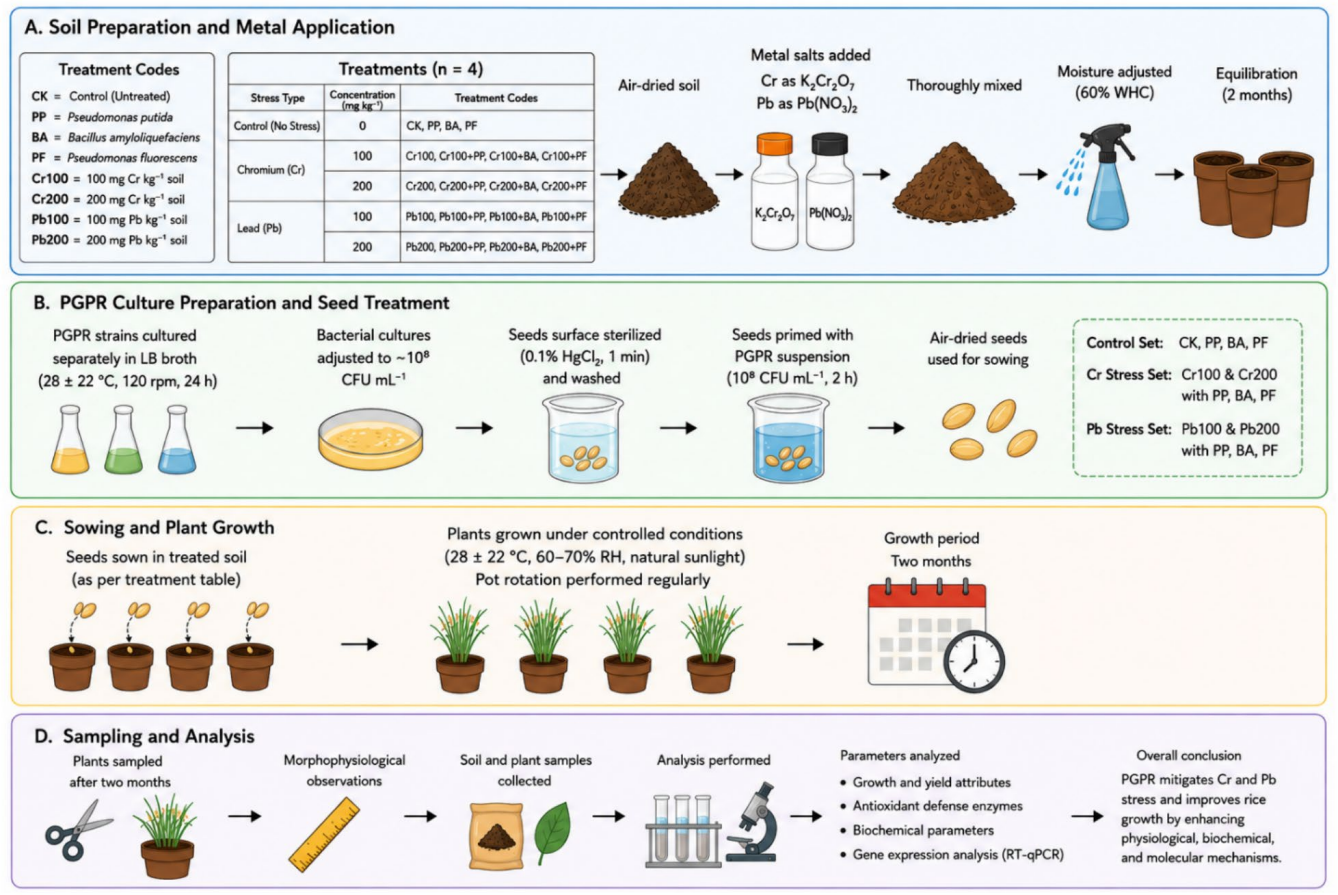
Experimental workflow illustrating soil preparation and stress application, PGPR inoculation and seed priming, and plant growth under controlled greenhouse conditions. Heavy metal stresses were applied using metal salts, while PGPR strains were cultured and used for seed priming (10⁸ CFU mL⁻^1^).

A pot experiment was conducted in the greenhouse facilities of the Department of Biological Sciences, College of Science, University of Jeddah, 21,493 Jeddah, Saudi Arabia. Pots were placed under glass house environment where they received natural sunlight, day/night humidity (60/70%) and day/night temperature (28/22 °C), respectively. “Sakha 101” rice cultivar was used as a test plant, and *O. sativa* seeds were obtained from the Department of Biological Sciences, College of Science, University of Jeddah, Saudi Arabia. Permission to conduct the greenhouse experiment and collect soil and plant materials was obtained from the Department of Biological Sciences, College of Science, University of Jeddah, Saudi Arabia, prior to the commencement of the study. Before sowing, the seeds were carefully washed and surface sterilized with 0.1% HgCl_2_ solution for 1 min to eliminate surface microbial contamination, followed by three thorough washes with distilled water to remove any residual HgCl_2_. Uncontaminated soil, obtained from the research field of Department of Biological Sciences, College of Science, University of Jeddah, Saudi Arabia, was air dried and passed through a 2-mm sieve. The initial physiochemical properties were as follow: pH (6.9), EC (0.9 dS cm⁻^1^), organic matter (17 g kg⁻^1^), extractable K (21 mg kg⁻^1^), total phosphorus (0.17 g kg⁻^1^), total nitrogen (1.6 g kg⁻^1^), cation exchange capacity (12.4 cmol kg⁻^1^), organic carbon (9.8 g kg⁻^1^), available Fe (4.6 mg kg⁻^1^), available Zn (1.8 mg kg⁻^1^). The soil used in the experiment was collected from a non-industrial research field area and was free from visible contamination prior to artificial Cr and Pb treatments. After contamination of soil with Cr using potassium dichromate (K₂Cr₂O₇) , and Pb as lead nitrate [Pb(NO₃)₂]^18^, the metals were applied individually at concentrations of 0, 100, and 200 mg kg⁻^1^ soil. Pots (30 cm × 40 cm) were filled with 10 kg of contaminated soil and subjected to four wetting–drying cycles over a two-month equilibration period to stabilize the distribution of Cr and Pb before sowing. Regarding the PGPR strains, i.e., *Pseudomonas putida*, *Bacillus amyloliquefaciens*, and *Pseudomonas fluorescens*, isolation and characterization were performed following the standard serial dilution and biochemical identification methods described by^19^. The selected PGPR strains were chosen based on their previously reported plant growth-promoting properties, including phytohormone production, nutrient mobilization, and stress tolerance capabilities reported in earlier studies^20^. The pots used in this study were rotated regularly in order to avoid environmental effects on the plants. A complete randomized design (CRD) with four replications was used. The detailed treatment combinations used in the present study are presented in Table 1. The total duration of experimental treatments was 3 months under controlled conditions. A detailed schematic presentation of the entire methodology is provided in Fig. 1.

**Table 1:** Experimental treatment combinations used to evaluate the effects of different PGPR strains on *Oryza sativa* under chromium (Cr) and lead (Pb) stress conditions.

Stress type	Treatment code	Description
Controls (No Stress)	CK	Absolute control (untreated)
	PP	*P. putida* alone
	BA	*B. amyloliquefaciens* alone
	PF	*P. fluorescens* alone
Chromium stress (100 mg kg^-1^)	Cr100	Cr (100 mg kg⁻^1^) alone
	Cr100 + PP	Cr (100 mg kg⁻^1^) + *P. putida*
	Cr100 + BA	Cr (100 mg kg⁻^1^) + *B. amyloliquefaciens*
	Cr100 + PF	Cr (100 mg kg⁻^1^) + *P. fluorescens*
Chromium stress (200 mg kg^-1^)	Cr200	Cr (200 mg kg⁻^1^) alone
	Cr200 + PP	Cr (200 mg kg⁻^1^) + *P. putida*
	Cr200 + BA	Cr (200 mg kg⁻^1^) + *B. amyloliquefaciens*
	Cr200 + PF	Cr (200 mg kg⁻^1^) + *P. fluorescens*
Lead stress (100 mg kg^-1^)	Pb100	Pb (100 mg kg⁻^1^) alone
	Pb100 + PP	Pb (100 mg kg⁻^1^) + *P. putida*
	Pb100 + BA	Pb (100 mg kg⁻^1^) + *B. amyloliquefaciens*
	Pb100 + PF	Pb (100 mg kg⁻^1^) + *P. fluorescens*
Lead stress (200 mg kg^-1^)	Pb200	Pb (200 mg kg⁻^1^) alone
	Pb200 + PP	Pb (200 mg kg⁻^1^) + *P. putida*
	Pb200 + BA	Pb (200 mg kg⁻^1^) + *B. amyloliquefaciens*
	Pb200 + PF	Pb (200 mg kg⁻^1^) + *P. fluorescens*

CK = control without metal stress and PGPR application; PP = *Pseudomonas putida*; BA = *Bacillus amyloliquefaciens*; PF = *Pseudomonas fluorescens*. Cr and Pb were applied individually at concentrations of 100 and 200 mg kg⁻^1^ soil.

### Inoculation with P. putida, B. amyloliquefaciens, and P. fluorescens

The PGPR strains, i.e., *P. putida*, *B. amyloliquefaciens*, and *P. fluorescens*, were cultured separately in LB broth at 28 °C for 24 h under continuous shaking to ensure proper bacterial growth. After incubation, the cultures were centrifuged to obtain bacterial pellets, which were subsequently dissolved in sterile distilled water to prepare bacterial suspensions with a concentration of 10^8^ CFU mL⁻^1^. *O. sativa* seeds were soaked separately in each bacterial suspension for 2 h prior to sowing to ensure effective colonization. Furthermore, the respective bacterial suspensions were applied to the soil of PGPR-treated pots at concentrations of 5 ppm and 10 ppm according to the treatment design. To maintain a stable bacterial population throughout the experimental period, fresh bacterial suspensions were reapplied to the soil at 15-day intervals. Although direct root colonization assays were not performed, repeated inoculation and maintenance of bacterial populations were carried out to ensure effective PGPR establishment during the experimental period. Fresh bacterial suspensions were reapplied to the soil at 15-day intervals to maintain bacterial populations throughout the experimental period. For the non-PGPR treatments, a mock inoculum was prepared following the same procedure used for the PGPR suspensions without the addition of bacterial pellets. Sterile distilled water was used instead of bacterial suspensions and was applied to the control pots at the same concentrations and time intervals as the PGPR treatments to maintain uniform experimental conditions across all treatments.

### Plant harvesting and data collection

After three months, remaining seedlings were up rooted and washed gently with the help of distilled water to eliminate the aerial dust and deposition. Functional leaf in each treatment was picked at a rapid growth stage during 09:00–10:30 a.m. The sampled leaves were washed with distilled water, immediately placed in liquid nitrogen, and stored in a freezer at − 80 °C for further analysis. All the harvested plants were divided into two parts i.e. roots and shoots to study different physio-biochemical traits. Leaves from each treatment group were picked for chlorophyll, carotenoid, oxidative stress and antioxidants analysis. Root and shoot lengths were measured straightway after the harvesting by using measuring scale and digital weighting balance to measure fresh biomass. Roots were uprooted and immersed in 20 mM Na_2_EDTA for 15–20 min to remove Cr and Pb adhered to the root surfaces. Then, roots were washed thrice with distilled water and finally once with de-ionized water and dried for further analysis. The different parts of the plant (roots and shoots) were oven-dehydrated at 65 °C for 72 h for Cr and Pb determination and the total plant dry weight was also measured.

### Determination of chlorophyll pigments and gas exchange attributes

For chlorophyll content analysis, 0.1 g of fresh leaf sample was extracted with 8 mL of 95% acetone for 24 h at 4 °C in the dark. The absorbance of the resultant solution was measured by a spectrophotometer (UV-2550; Shimadzu, Kyoto, Japan) at 646.6, 663.6, and 450 nm. The chlorophyll content was then calculated using the standard method described by^21^, ensuring that the final volume of each sample, after adding the supernatant to 85% acetone, was adjusted to 10 mL.

On the same days, gaseous exchange was also measured. Net photosynthesis (*Pn*), leaf stomatal conductance (*Gs*), transpiration rate (*Ts*), and intercellular carbon dioxide concentration (*Ci*) were measured from three different plants in each treatment group. Measurements were taken from 9:00 a.m. to 11:00 a.m. to ensure consistent environmental conditions with a clear sky. Rates of leaf *Pn*, *Gs*, *Ts*, and *Ci* were measured with an LI-COR gas-exchange system (LI6400; LICOR Biosciences, Lincoln, NE, USA) with a redblue LED light source on the leaf chamber. In the LI-COR cuvette, CO_2_ concentration was set as 380 µmol mol^−1^ and LED light intensity was set at 1000 µmol m^−2^ s^−1^, which is the average saturation intensity for photosynthesis in *O. sativa*.

### Evaluation of oxidative stress biomarkers

The degree of lipid peroxidation was evaluated as malondialdehyde (MDA) content. Briefly, 0.1 g of frozen leaves were ground at 4 °C in a mortar with 25 mL of 50 mM phosphate buffer solution (pH 7.8) containing 1% polyethene pyrrole. The homogenate was centrifuged at 10,000 × g at 4 °C for 15 min. The mixtures were heated at 100 °C for 15–30 min and then quickly cooled in an ice bath. The absorbance of the supernatant was recorded by using a spectrophotometer (xMark™ microplate absorbance spectrophotometer; BioRad, United States) at wavelengths of 532, 600 and 450 nm. Lipid peroxidation was expressed as l mol g − 1 using the following formula: 6.45 (A532 − A600) − 0.56 A450. Lipid peroxidation was measured using a method previously published by^22^.

For hydrogen peroxide (H_2_O_2_) assay, leaf and root samples were homogenized with 50 mM phosphate buffer at pH 6.5. After that, homogenized samples were centrifuge at 6000 × g for 25 min, followed by the addition of H_2_SO_4_ (20% v/v) and again centrifuged at 6000 × g for 15 min. H_2_O_2_ contents were estimated by taking the absorbance at 410 nm and calculations were completed with the help of extinction coefficient (0.28 µmol − ^1^ cm − ^1^)^23^.

### Evaluation of enzymatic antioxidant defense system

To evaluate enzyme activities, fresh leaves (0.5 g) were homogenized in liquid nitrogen and 5 mL of 50 mmol sodium phosphate buffer (pH 7.0), including 0.5 mmol EDTA and 0.15 mol NaCl. The homogenate was centrifuged at 12,000 × g for 10 min at 4 °C, and the supernatant was used for the measurement of superoxidase dismutase (SOD) and peroxidase (POD) activities. SOD activity was assayed in 3 mL reaction mixture containing 50 mM sodium phosphate buffer (pH 7), 56 mM nitro blue tetrazolium, 1.17 mM riboflavin, 10 mM methionine, and 100 µL enzyme extract. Finally, the sample was measured by using a spectrophotometer (xMark™ microplate absorbance spectrophotometer; Bio-Rad). Enzyme activity was measured using a method by^24^.

Peroxidase activity in the leaves and roots was estimated using the method of^25^ using guaiacol as the substrate. A reaction mixture (3 mL) containing 0.05 mL of enzyme extract, 2.75 mL of 50 mM phosphate buffer (pH 7.0), 0.1 mL of 1% H_2_O_2_ and 0.1 mL of 4% guaiacol solution was prepared. Increases in the absorbance at 470 nm because of guaiacol oxidation was recorded for 2 min. One unit of enzyme activity was defined as the amount of the enzyme.

Catalase (CAT) activity was analyzed according to^26^. The assay mixture (3.0 mL) was comprised of 100 µL enzyme extract, 100 µL H_2_O_2_ (300 mM) and 2.8 mL 50 mM phosphate buffer with 2 mM ETDA (pH 7.0). The CAT activity was measured from the decline in absorbance at 240 nm as a result of H_2_O_2_ loss (ε = 39.4 mM − ^1^ cm − ^1^).

Ascorbate peroxidase (APX) activity was measured according to^23^. The mixture containing 100 µL enzyme extract, 100 µL ascorbate (7.5 mM), 100 µL H_2_O_2_ (300 mM), and 2.7 mL 25 mM potassium phosphate buffer with 2 mM EDTA (pH 7.0) was used for measuring APX activity. The oxidation pattern of ascorbate was estimated from the variations in wavelength at 290 nm (ε = 2.8 mM − ^1^ cm − ^1^).

Quantitative real-time PCR (RT-qPCR) assay was applied to investigate the expression levels of 4 stress-related genes, including Fe-SOD, POD, CAT and APX. Total RNA was extracted from leaf tissue samples using RNeasy Plant Mini kits (Qiagen, Manchester, UK). The RT-qPCR was performed using an Applied Biosystems 7500 Fast Real-Time PCR System with the following cycling parameters: initial denaturation at 95 °C for 5 min, followed by 40 cycles of denaturation at 95 °C for 15 s, annealing at 58–60 °C (optimized for each primer pair) for 30 s, and extension at 72 °C for 30 s. A melting curve analysis was conducted from 65 °C to 95 °C to confirm the specificity of the amplification. Contaminating DNA was then removed and first-strand cDNAs were prepared using Reverse Transcription kits (Qiagen, Manchester, UK). RT-qPCR analysis was conducted as reported in the protocol of QuantiTect SYBR Green PCR kit (Qiagen, Manchester, UK). Reaction volume and PCR amplification conditions were adjusted as mentioned by^27^.

### Evaluation of non-enzymatic metabolites and sugar accumulation

Plant ethanol extracts were prepared for the determination of non-enzymatic antioxidants and some key osmolytes. For this purpose, 50 mg of dry plant material was homogenized with 10 mL ethanol (80%) and filtered through Whatman No. 41 filter paper. The residue was re-extracted with ethanol, and the 2 extracts were pooled together to a final volume of 20 mL. The determination of flavonoids^28^, phenolics^29^, ascorbic acid^30^, anthocyanin^31^, and total sugars^32^ was performed from the extracts. Fresh leaf material (0.1 g) was mixed thoroughly in 5 mL aqueous sulphosalicylic acid (3%). The mixture was centrifuged at 10,000 × g for 15 min, and an aliquot (1 mL) was poured into a test tube having 1 mL acidic ninhydrin and 1 mL glacial acetic acid. The reaction mixture was first heated at 100 °C for 10 min and then cooled in an ice bath. The reaction mixture was extracted with 4 mL toluene, and test tubes were vortexed for 20 s and cooled. Thereafter, the light absorbance at 520 nm was measured by using a UV–VIS spectrophotometer (Hitachi U-2910, Tokyo, Japan). The free proline content was determined on the basis of the standard curve at 520 nm absorbance.

### Evaluation of proline metabolic responses

To measure proline concentrations, 0.5 g of shoot tissues were ground in sulfosalicylic acid and then centrifuged, and the supernatant was collected from each sample. The proline concentration in each sample was measured^33^. Specifically, the supernatant from each sample was reacted with acid ninhydrin, and the resulting colorimetric reaction was measured to determine the proline concentration by “UV-1700 pharmaSpec spectrophotometer”. The ProDH “proline dehydrogenase”, P5CR “pyrroline5-carboxylate reductase”, and P5C “pyrroline-5-carboxylate” were measured using kits provided by Jiangsu Meibiao Biological Technology Co., Ltd. Enzyme activities were accurately measured using these reagent kits, which include all chemicals and related instructions by “UV-1700 pharmaSpec spectrophotometer”.

### Evaluation of the AsA–GSH antioxidant cycle

Glutathione (GSH), glutathione disulfide (GSSH), DHA (dehydroascorbic acid), and AsA were determined in fresh leaves^34^ and were extracted by homogenizing 0.2 g of leaves in TCA and then collecting the supernatant by centrifugation. GSH concentration was measured in a solution including phosphate buffer, supernatant, and DTNB reagent (PBS, pH 7.0). The amount of GSH was determined by a spectrophotometer. To measure the AsA content, NaH_2_PO_4_ solution, enzyme extract, distilled water, and 10% TCA were mixed to determine the concentration of AsA in the samples. After a 30-s incubation period, FeCl_3_ solution, H_3_PO_4_, and 2,2′ -dipyridine were added to the reaction mixture. The FeCl_3_ and 2,2′ -dipyridine reacted with the AsA to produce a red-colored complex that can be measured spectrophotometrically at 525 nm. The amount of AsA present in the sample was calculated.

### Evaluation of cell wall component distribution

Cell wall isolation was done as reported by^35^. Shoots (4 g) were placed in a mortar and ground with liquid nitrogen. The homogenized samples were transferred to centrifuge tubes and 75% ethanol was added and incubated at 25 °C. The samples were centrifuged. The bottom sediment was further homogenized in 10 mL of each of acetone, chloroform, and methanol (v: v = 1:1) for 30 min each, with shaking at room temperature. The homogenate was centrifuged. The remaining cell wall components were lyophilized until dry sediment was obtained. The lyophilized cell wall components were analyzed for biochemical assays. Subsequently, the separation of the hemicellulose fraction was carried out. Approximately 3 mg CW was mixed with water in an Eppendorf tube. The mixture was boiled for 1 h using a heating block or hot plate set at 100 °C and centrifuged. The above procedure was repeated for duplicate samples. After 12 h, the precipitate was extracted twice with 1 mL of KOH (24%, w/v) at room temperature. After each extraction, centrifugation was done. The hemicellulose concentration was measured at 540 nm absorbance.

Pectin Assay Kit was used to detect pectin. Pectinesterase Assay Kit was used to detect PME activity. The Cellulose Assay Kit was used to detect cellulose concentrations using kits provided by Jiangsu Meibiao Biological Technology Co., Ltd. Enzyme activities were accurately measured using these reagent kits, which include all chemicals and related instructions. DM was calculated using the formula: demethylation degree = 100-DM, where DM is the degree of methylation. 

### Evaluation of Cr and Pb Uptake and nutrient status

Finely ground samples were digested with pure HNO_3_ at 190 °C for 45 min (10 min pre-heating, 15 min heating, 20 min cooling) in a microwave oven (Mars 6, CEM Corporation, USA) with the settings described in details by^36^. Samples were diluted with 2% HNO_3_ and determined by inductively coupled plasma-mass spectroscopy (ICP-MS; Agilent 7700, Agilent Technologies Inc., USA).

Essential nutrients such as calcium (Ca^2+^), magnesium (Mg^2+^), iron (Fe^2+^), and phosphorus (P) analysis, plant shoots were washed twice in redistilled water, dipped in 20 mM EDTA for 3 s and then, again washed with deionized water twice for the removal of adsorbed metal on the plant surface. The washed samples were then oven dried for 24 h at 105 °C. The dried roots and shoots were digested using the wet digestion method in HNO_3_: HClO_4_ (7:3 V/V) until clear samples were obtained. Each sample was filtered and diluted with redistilled water up to 50 mL. The shoot contents of Ca^2+^, Mg^2+^, Fe^2+^, and P were analyzed using the Atomic Absorption Spectrophotometer (AAS) model Agilent 240FS-AA. 

### Statistical analysis

 All experimental data were expressed as arithmetic means and analyzed using Statistix 8.1 software. The experiment was conducted using a completely randomized design (CRD) with four biological replicates. Metal stress levels and PGPR treatments were considered as independent variables, while morphophysiological, biochemical, antioxidant, and molecular parameters were treated as dependent variables. Prior to analysis, the data were tested for normality and homogeneity of variance. Statistical analyses were performed to evaluate treatment effects among different metal stress levels and PGPR applications. Mean comparisons among treatment combinations were conducted using analysis of variance (ANOVA) followed by Tukey’s HSD test at P ≤ 0.05. Graphical representations were prepared using Origin-2017.

## Results

### Effects of PGPR on growth, biomass, and photosynthetic attributes of O. sativa under metal stress

**Fig. 2:**
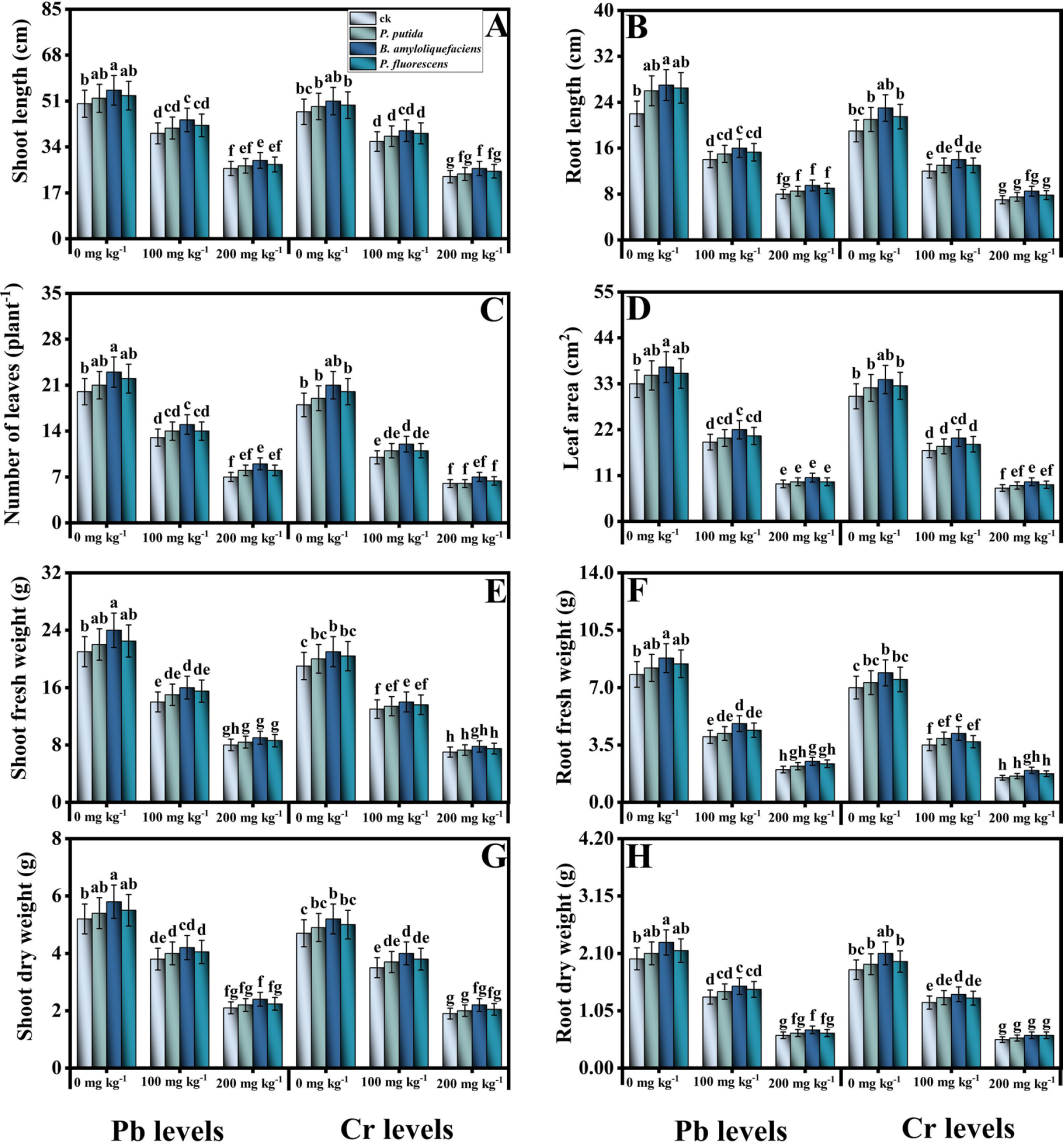
Effects of *Pseudomonas putida*, *Bacillus amyloliquefaciens*, and *Pseudomonas fluorescens* on morphophysiological attributes of *Oryza sativa* grown under different Pb and Cr stress levels. (**A**) shoot length, (**B**) root length, (**C**) number of leaves, (**D**) leaf area, (**E**) shoot fresh weight, (**F**) root fresh weight, (**G**) shoot dry weight, and (**H**) root dry weight. Bars represent mean ± standard deviation (SD) of four replicates. Different lowercase letters indicate statistically significant differences among treatment combinations according to ANOVA followed by Tukey’s HSD test at *P* ≤ 0.05. CK = control without PGPR inoculation.

**Fig. 3:**
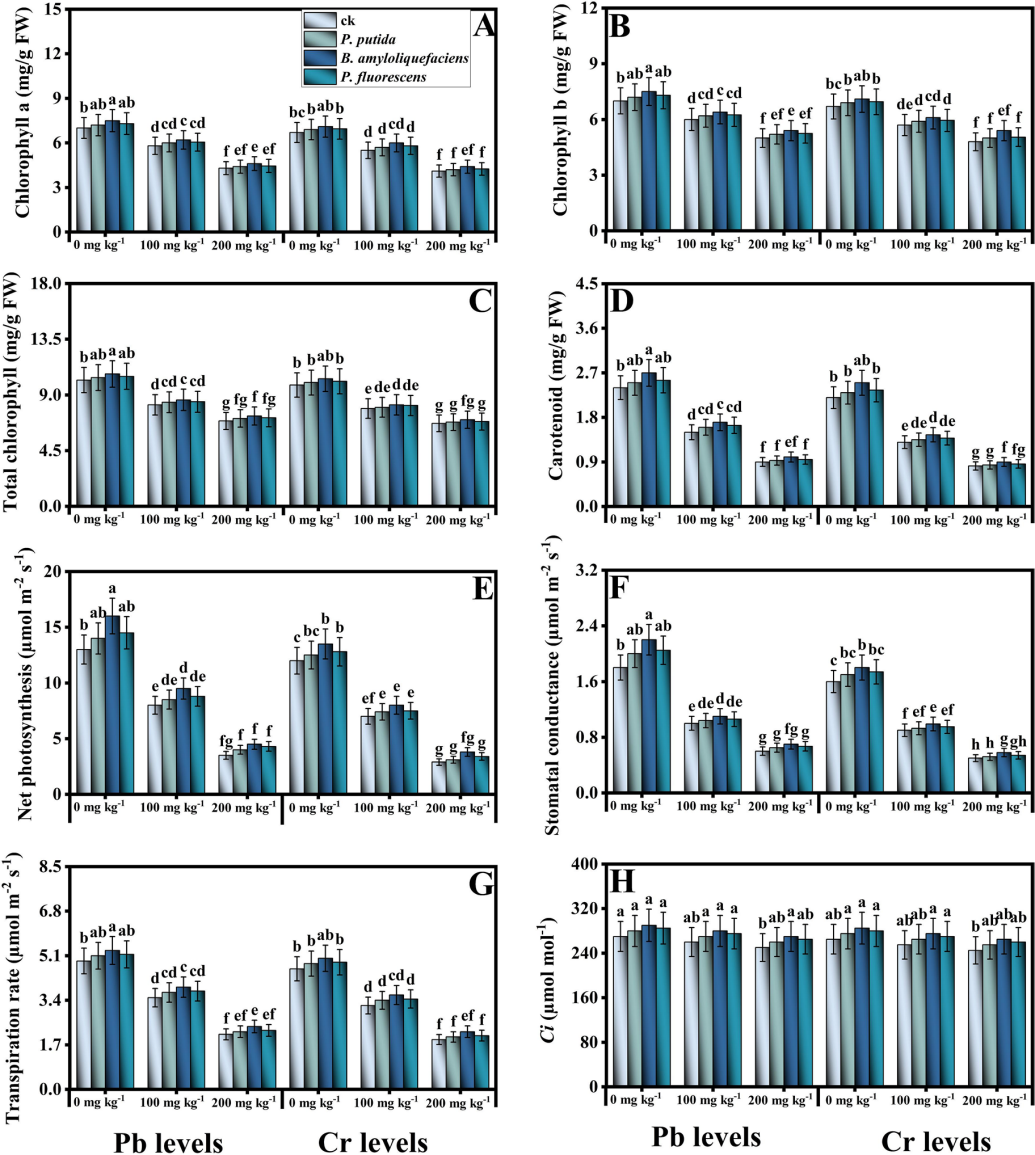
Effects of *Pseudomonas putida*, *Bacillus amyloliquefaciens*, and *Pseudomonas fluorescens* on photosynthetic pigments and gas exchange parameters of *Oryza sativa* grown under different Pb and Cr stress levels. (**A**) chlorophyll a, (**B**) chlorophyll b, (**C**) total chlorophyll, (**D**) carotenoid content, (**E**) net photosynthesis, (**F**) stomatal conductance, (**G**) transpiration rate, and (**H**) intercellular CO₂ concentration (Ci). Bars represent mean ± standard deviation (SD) of four replicates. Different lowercase letters indicate statistically significant differences among treatment combinations according to ANOVA followed by Tukey’s HSD test at *P* ≤ 0.05. CK = control without PGPR inoculation.

In the present study, various growth parameters and photosynthetic pigments and also the gas exchange parameters in rice (*Oryza sativa* L.) under the chromium (Cr) and lead (Pb) stress with the application of *Pseudomonas putida*, *Bacillus amyloliquefaciens*, and *Pseudomonas fluorescens* were measured. Growth and biomasses of *O. sativa* are presented in Fig. 2, while gas exchange attributes are presented in Fig. 3. Specifically, we observed significant (*P* ≤ 0.05) decrease in root length, shoot length, number of leaves, leaf area, shoot fresh weight, root fresh weight, shoot dry weight, and root dry weight, as well as reductions in chlorophyll a, chlorophyll b, total chlorophyll, carotenoid content, net photosynthesis, stomatal conductance, transpiration rate, and intercellular CO_2_ (Figs. 2 and 3). However, the application of *P. putida*, *B. amyloliquefaciens*, and *P. fluorescens* significantly improved root and shoot growth, biomass accumulation, photosynthetic pigments, and gas exchange attributes compared with non-inoculated plants under Cr and Pb stress conditions. Among the tested PGPR strains, *B. amyloliquefaciens* showed comparatively better improvement in most morphophysiological traits under severe metal stress.

### PGPR-mediated regulation of oxidative stress and antioxidant defense mechanisms in O. sativa

**Fig. 4:**
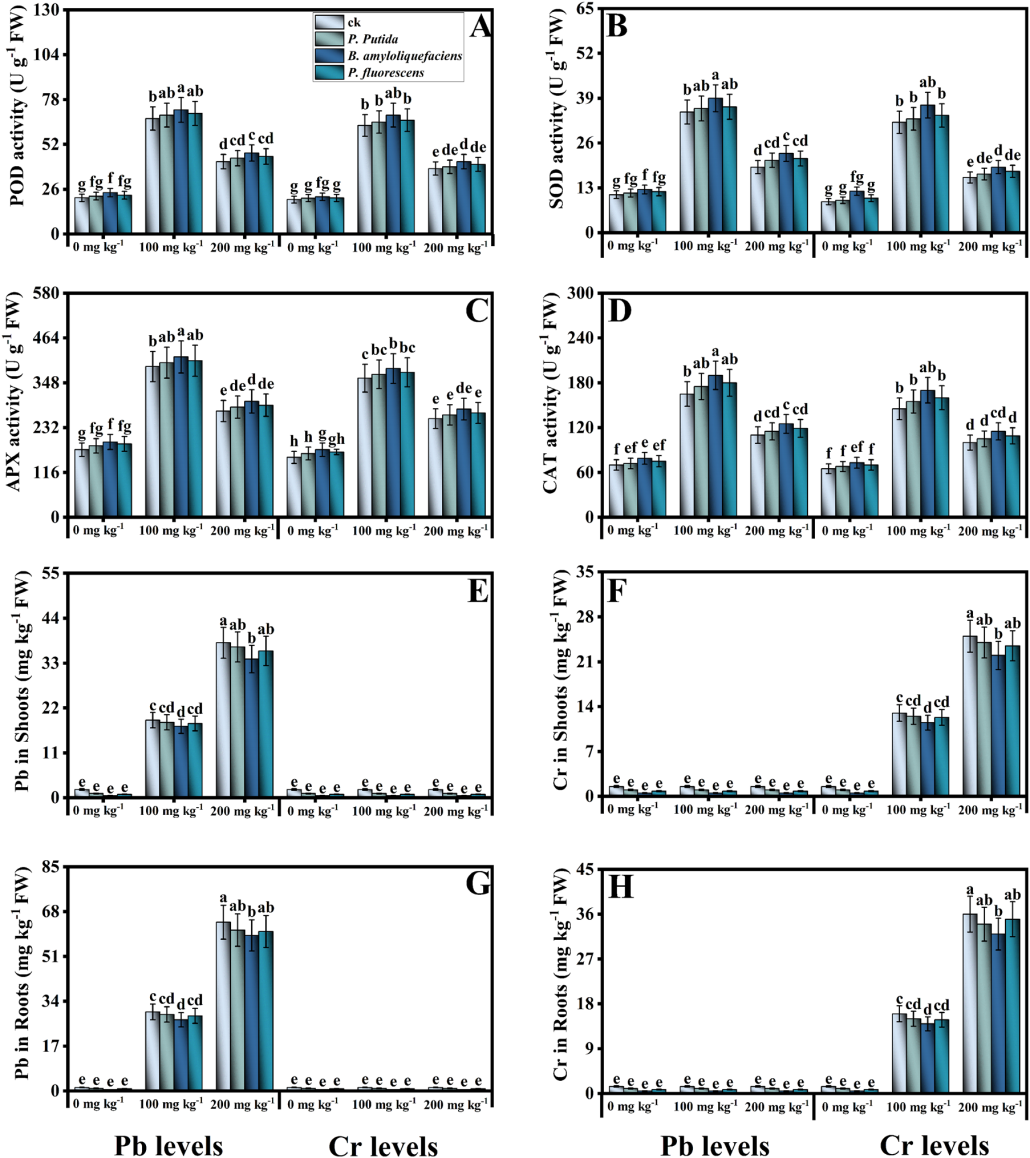
Effects of *Pseudomonas putida*, *Bacillus amyloliquefaciens*, and *Pseudomonas fluorescens* on antioxidant enzyme activities and Cr/Pb accumulation in *Oryza sativa* grown under different Pb and Cr stress levels. (**A**) POD activity, (**B**) SOD activity, (**C**) APX activity, (**D**) CAT activity, (**E**) Pb content in shoots, (**F**) Cr content in shoots, (**G**) Pb content in roots, and (**H**) Cr content in roots. Bars represent mean ± standard deviation (SD) of four replicates. Different lowercase letters indicate statistically significant differences among treatment combinations according to ANOVA followed by Tukey’s HSD test at *P* ≤ 0.05. CK = control without PGPR inoculation.

**Fig. 5:**
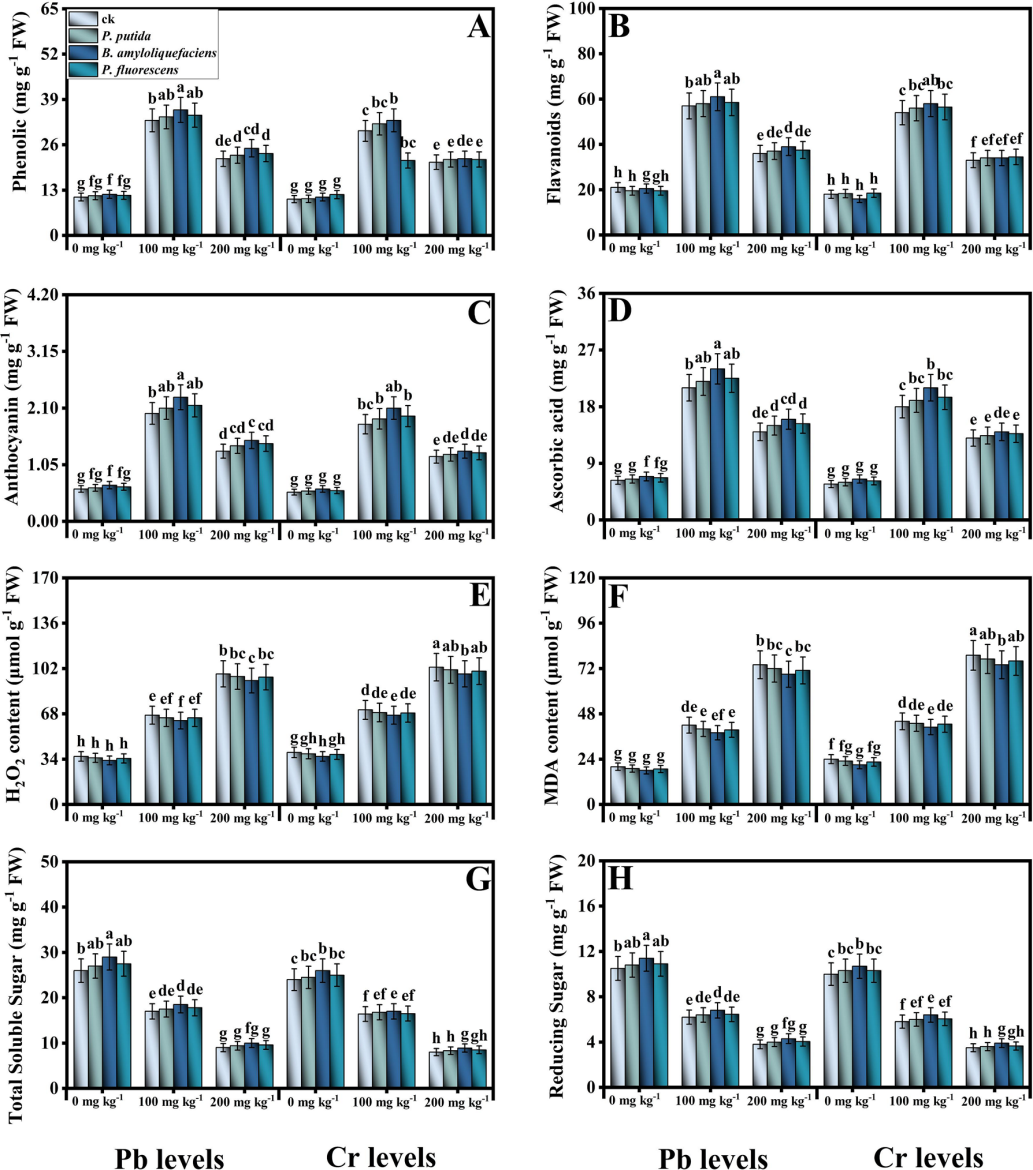
Effects of *Pseudomonas putida*, *Bacillus amyloliquefaciens*, and *Pseudomonas fluorescens* on non-enzymatic compounds, oxidative stress biomarkers, and sugar contents in *Oryza sativa* grown under different Pb and Cr stress levels. (**A**) phenolic content, (**B**) flavonoid content, (**C**) anthocyanin content, (**D**) ascorbic acid content, (**E**) H₂O₂ content, (**F**) MDA content, (**G**) total soluble sugar, and (**H**) reducing sugar. Bars represent mean ± standard deviation (SD) of four replicates. Different lowercase letters indicate statistically significant differences among treatment combinations according to ANOVA followed by Tukey’s HSD test at *P* ≤ 0.05. CK = control without PGPR inoculation.

**Fig. 6:**
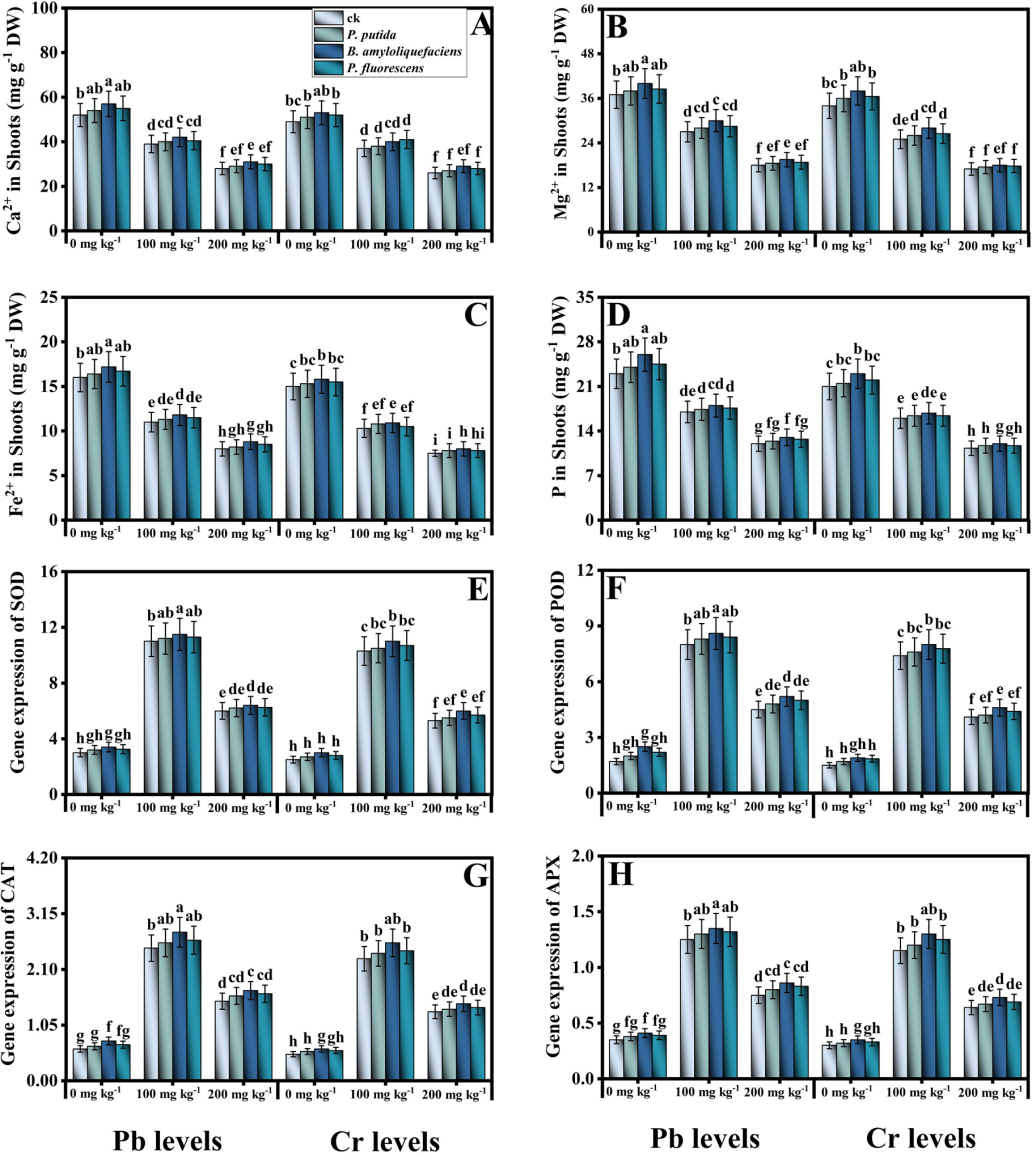
Effects of *Pseudomonas putida*, *Bacillus amyloliquefaciens*, and *Pseudomonas fluorescens* on nutrient accumulation and antioxidant-related gene expression in *Oryza sativa* grown under different Pb and Cr stress levels. (**A**) Ca^2^⁺ content in shoots, (**B**) Mg^2^⁺ content in shoots, (**C**) Fe^2^⁺ content in shoots, (**D**) P content in shoots, (**E**) gene expression of SOD, (**F**) gene expression of POD, (**G**) gene expression of CAT, and (**H**) gene expression of APX. Bars represent mean ± standard deviation (SD) of four replicates. Different lowercase letters indicate statistically significant differences among treatment combinations according to ANOVA followed by Tukey’s HSD test at *P* ≤ 0.05. CK = control without PGPR inoculation.

In the present study, different oxidative stress biomarkers, i.e., malondialdehyde (MDA) and hydrogen peroxide (H_2_O_2_) were measured from the leaves of *O. sativa* as presented in (Fig. 5). According to the results, it was observed that the Cr and Pb stress caused a significant (*P* ≤ 0.05) increase in MDA, and H_2_O_2_ content in the plants compared to the plants which were not grown in the toxic levels of Cr and Pb stress in the nutrient solution. Although the application of *P. putida*, *B. amyloliquefaciens*, and *P. fluorescens* decreases the MDA, and H_2_O_2_ content in *O. sativa* under the studied level of Cr and Pb toxicity in the nutrient solution. Different enzymatic antioxidants, i.e., superoxidase dismutase (SOD), ascorbate peroxidase (APX), peroxidase (POD), catalase (CAT), and non-enzymatic compounds, i.e., phenolic, anthocyanin, flavonoids, ascorbic acid, and also their relevant gene expression, i.e., SOD, POD, CAT, and APX were also measured from *O. sativa*. The results regarding the enzymatic antioxidants are presented in Fig. 4,5 and their specific gene expressions are presented in Fig. 6, and the results regarding the nonenzymatic compounds are presented in Fig. 5. According to the results, we have noticed that the Cr and Pb toxicity in the nutrient solution significantly increases the enzymatic antioxidants, i.e., SOD, POD, CAT, and APX and their relevant gene expression and also the non-enzymatic compounds, i.e., phenolic, anthocyanin, flavonoids, ascorbic acid compared to the plants grown without the toxic concentration of Cr and Pb toxicity in the nutrient solution. Present findings also showed that the application of *P. putida*, *B. amyloliquefaciens*, and *P. fluorescens* also increases the activity of SOD, POD, CAT, and APX and their relevant gene expression and also the non-enzymatic compounds, i.e., phenolic, anthocyanin, flavonoids, ascorbic acid, compared to the plants which were not treatment with the application of *P. putida*, *B. amyloliquefaciens*, and *P. fluorescens*. In addition, the maximum activity of SOD, POD, CAT, and APX and their relevant gene expression and also the non-enzymatic compounds, i.e., phenolic, anthocyanin, flavonoids, ascorbic acid, were observed in the plants which are grown in the application *P. putida*, *B. amyloliquefaciens*, and *P. fluorescens* under the toxic levels of Cr and Pb concentration in the nutrient solution. Overall, PGPR-treated plants exhibited improved antioxidant defense capacity and reduced oxidative damage under Cr and Pb stress, with *B. amyloliquefaciens* showing relatively stronger stimulatory effects on antioxidant activities and related gene expression.

### Effects of PGPR on nutrient acquisition and heavy metal uptake in O. sativa

The results regarding the treatment of Cr and Pb stress (100 and 200 mg kg⁻^1^) in the nutrient solution and seed inoculation with different PGPR strains, i.e., *P. putida, B. amyloliquefaciens, and P. fluorescens* on Cr and Pb uptake in the roots and shoots of *O. sativa* is presented in Fig. 4. According to the given results, we have noticed that toxic concentration of Cr and Pb in the nutrient solution (100 and 200 mg kg⁻^1^) induced a significant (*P* ≤ 0.05) increase in the content of Cr and Pb concentration in the roots and shoots of *O. sativa*, but significant (*P* ≤ 0.05) decreased in the essential nutrient contents i.e., calcium (Ca^2+^), magnesium (Mg^2+^), iron (Fe^2+^) and phosphorus (P) compared with plants that were grown in the soil treated with 0 mg kg⁻^1^ of Cr and Pb in the nutrient solution (Fig. 6). The results also illustrated that the application of different types of PGPR strains, i.e., *P. putida, B. amyloliquefaciens, and P. fluorescens* decreased the content of Cr and Pb concentration in the roots and shoots of *O. sativa*, while increased the concentration of essential minerals i.e., Ca^2+^, Mg^2+^, Fe^2+^, P compared with plants that were grown without the application with the different types of PGPR i.e., *P. putida, B. amyloliquefaciens, and P. fluorescens*. Among the tested strains, PGPR inoculation effectively reduced Cr and Pb accumulation while improving mineral nutrient acquisition in *O. sativa* under metal stress conditions.

### Effects of PGPR on proline metabolism, ASA–GSH cycle, and cell wall fractionation under metal stress

**Fig. 7:**
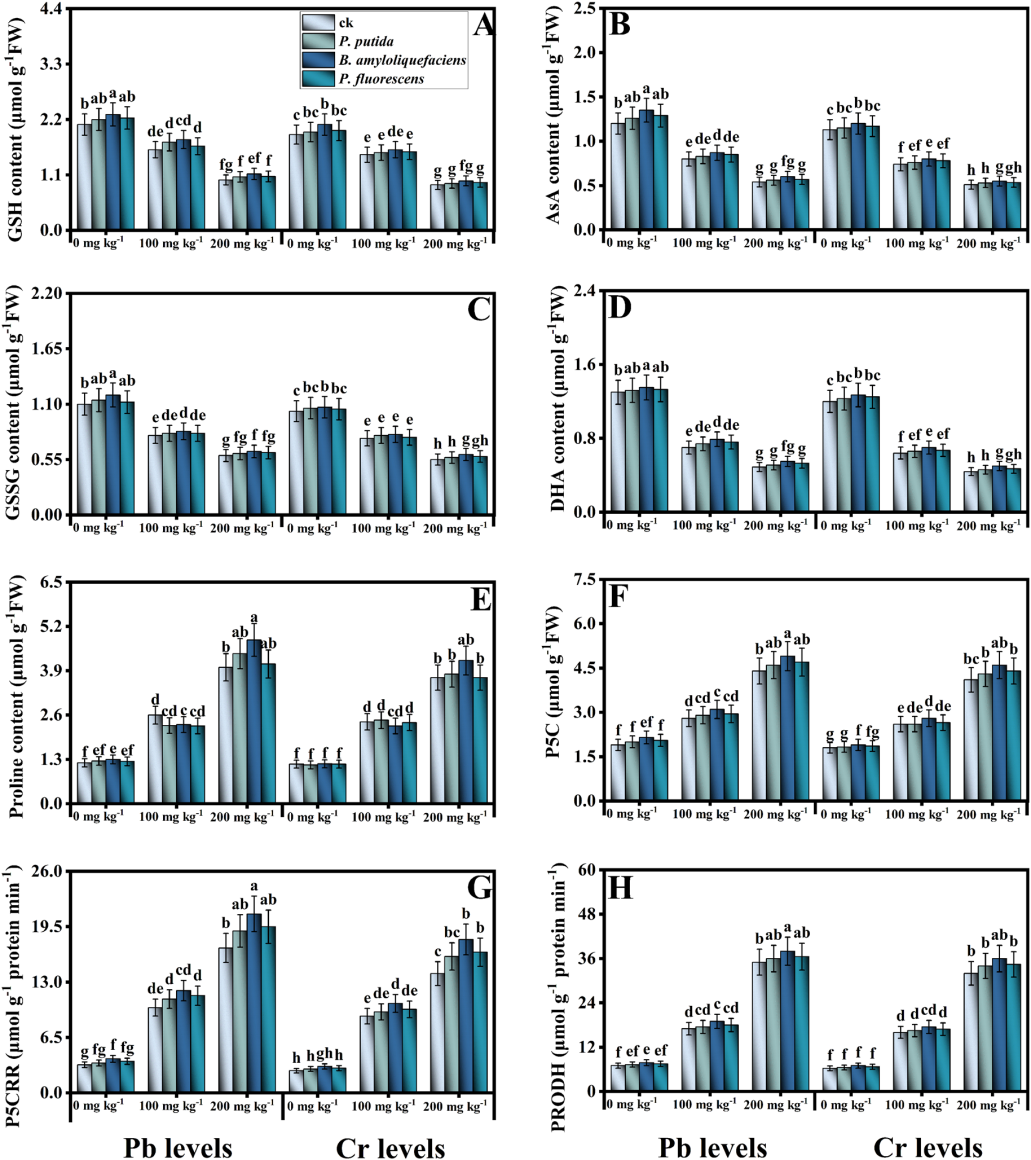
Effects of *Pseudomonas putida*, *Bacillus amyloliquefaciens*, and *Pseudomonas fluorescens* on AsA–GSH cycle components and proline metabolism in *Oryza sativa* grown under different Pb and Cr stress levels. (**A**) GSH content, (**B**) AsA content, (**C**) GSSG content, (**D**) DHA content, (**E**) proline content, (**F**) P5C content, (**G**) P5CR activity, and (**H**) PRODH activity. Bars represent mean ± standard deviation (SD) of four replicates. Different lowercase letters indicate statistically significant differences among treatment combinations according to ANOVA followed by Tukey’s HSD test at *P* ≤ 0.05. CK = control without PGPR inoculation.

In the present study, total soluble sugar and reducing sugar contents were also measured in O. sativa under Cr and Pb stress and are presented in Fig. 5. The proline-related attributes and AsA–GSH cycle components are presented in Fig. 7. The proline-related parameters, including proline, pyrroline-5-carboxylate (P5C), pyrroline-5-carboxylate reductase (P5CR), and pyrroline-5-carboxylate dehydrogenase (PRODH), were significantly affected by Cr and Pb stress. The results showed significant variations in proline metabolism under metal stress conditions compared with the control plants. However, the application of *P. putida*, *B. amyloliquefaciens*, and *P. fluorescens* further modulated the proline-related parameters in *O. sativa* under Cr and Pb stress conditions. Similarly, components of the AsA–GSH cycle, including glutathione (GSH), ascorbate (AsA), glutathione disulfide (GSSG), and dehydroascorbic acid (DHA), were also significantly influenced by Cr and Pb stress (Fig. 7). PGPR application further regulated the contents of GSH, AsA, GSSG, and DHA compared with untreated stressed plants, indicating improved antioxidant and redox regulation under metal stress conditions.

**Fig. 8:**
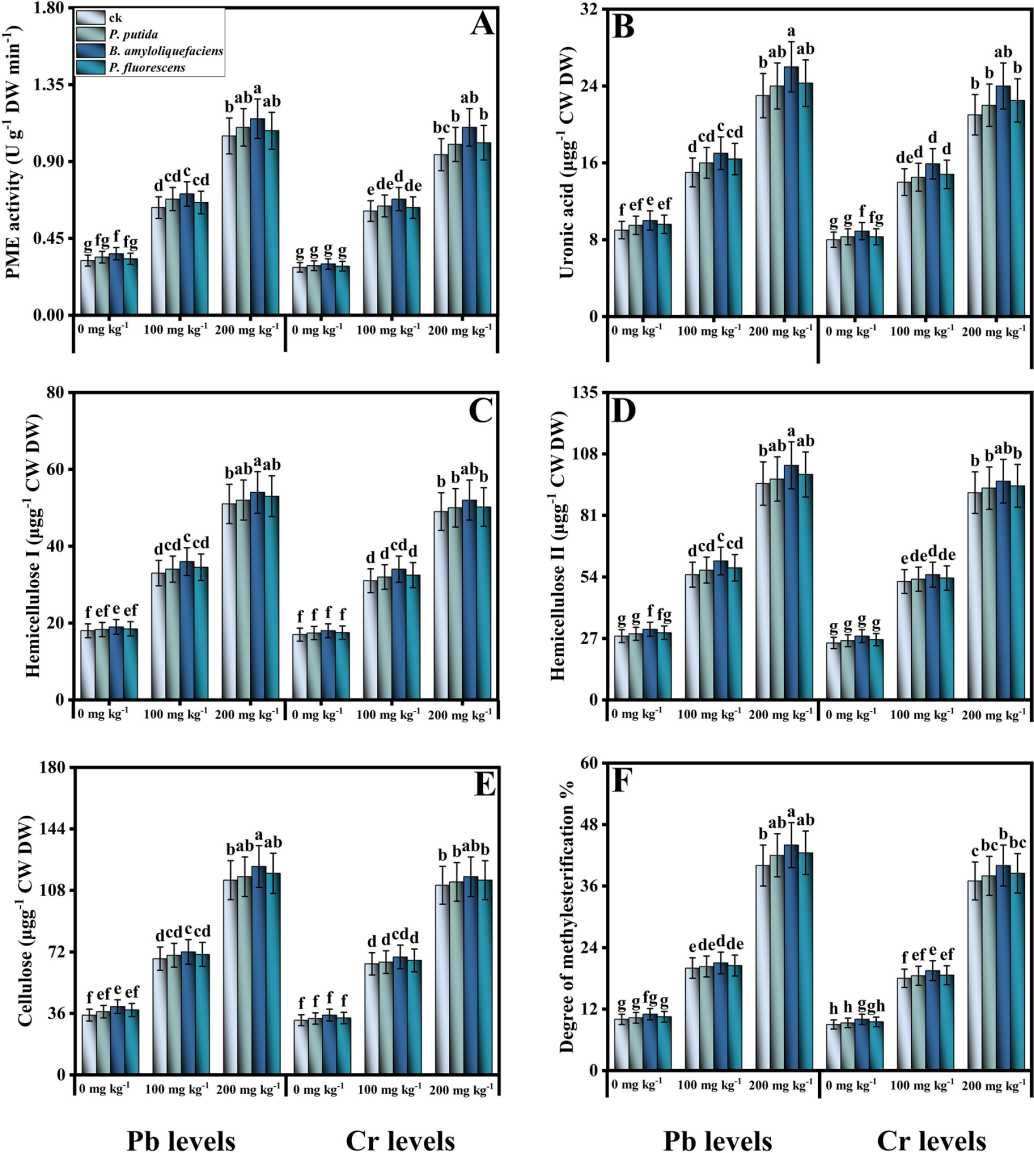
Effects of *Pseudomonas putida*, *Bacillus amyloliquefaciens*, and *Pseudomonas fluorescens* on cell wall component fractionation and structural modifications in *Oryza sativa* grown under different Pb and Cr stress levels. (**A**) PME activity, (**B**) uronic acid content, (**C**) hemicellulose I content, (**D**) hemicellulose II content, (**E**) cellulose content, and (**F**) degree of methylesterification. Bars represent mean ± standard deviation (SD) of four replicates. Different lowercase letters indicate statistically significant differences among treatment combinations according to ANOVA followed by Tukey’s HSD test at *P* ≤ 0.05. CK = control without PGPR inoculation.

Cellular compartment fractionation, i.e., pectin methylesterase activity, uronic acid, hemicellulose I, hemicellulose II, cellulose, and pectin methylesterase was also determined from the *O. sativa* and is presented as Fig. 8. Results from the present study showed that the Cr and Pb toxicity causes a significant (*P* ≤ 0.05) increase in the pectin methylesterase activity, uronic acid, hemicellulose I, hemicellulose II, cellulose, and pectin methylesterase when compared to the control. However, the application of *P. putida, B. amyloliquefaciens, and P. fluorescens* further increases the content of pectin methylesterase activity, uronic acid, hemicellulose I, hemicellulose II, cellulose, and pectin methylesterase in *O. sativa*. Overall, PGPR application improved osmotic regulation, antioxidant metabolism, and cell wall-associated defense mechanisms under Cr and Pb stress conditions.

## Discussion

Heavy metals including chromium (Cr) and lead (Pb) are recognized as important environmental toxic factors that adversely affect plant growth and yield^37,38^. Consequently, plant growth and biomass production have become pivotal morphological indicators under Cr and Pb-stressed environments^39^. It has been previously reported that Cr and Pb stress negatively affects plant biomass and photosynthetic efficiency in different plant species, depending upon several factors including plant species, dose, and duration of Cr and Pb exposure^39,40^. Chromium and Pb disrupt cell membrane integrity, inhibit enzymatic activities, and impair cell division and elongation, ultimately leading to stunted root and shoot growth^7,41,42^. Photosynthetic efficiency also declines due to Cr and Pb-induced chlorophyll degradation and disruption of the photosynthetic machinery^43–45^. When plants are exposed to excessive Cr and Pb concentrations, these metals compete with essential mineral ions for uptake, resulting in deficiencies of nutrients such as Ca, Mg, Fe, and P^18^. Excessive ROS generation under Cr and Pb stress overwhelms the plant antioxidant defense system, leading to alterations in SOD, POD, CAT, and APX activities, along with disturbances in proline metabolism and the AsA–GSH cycle^40,46–48^. It is well documented that Cr and Pb toxicity induces oxidative injury in plants through excessive ROS generation associated with Fenton and Haber–Weiss reactions, which further aggravates cellular damage^49–51^. The plant cell wall plays a crucial role in the development of stress tolerance against metals and other environmental stresses. Moreover, the cell wall forms a rigid structural barrier that restricts the entry of toxic ions into the cytoplasm^52,53^. Polysaccharides present in the cell wall, including cellulose, hemicellulose, and pectin, undergo significant modifications in response to environmental stress conditions^54–56^. For instance, pectin methylesterase can alter the degree of pectin methylation, thereby influencing the binding capacity of metal ions to the cell wall. In addition, Cr and Pb stress promotes lignin accumulation within the cell wall, resulting in alterations in its chemical composition and structural properties^52,57,58^. In the present study, Cr was applied as potassium dichromate, which primarily represents Cr(VI). However, Cr speciation in soil may change during the equilibration and incubation period depending on soil physicochemical properties, microbial activity, and redox conditions. Previous studies have reported that Cr(VI) can undergo partial reduction to the relatively less mobile and less toxic Cr(III) form in soil environments, which may influence its bioavailability and phytotoxicity^59,60^. Although Cr speciation and redox transformations were not directly determined in the present investigation, these processes may have contributed to the observed variations in Cr uptake and stress responses in *O. sativa*.

*Pseudomonas putida, Bacillus amyloliquefaciens, and Pseudomonas fluorescens* are well known PGPR for their beneficial roles in improving plant growth and stress tolerance under adverse environmental conditions^61–63^. The present findings are consistent with previous studies reporting that PGPR significantly improve plant growth and biomass under heavy metal stress through enhanced nutrient acquisition, phytohormone production, and improved root architecture^64,65^. These PGPR may contribute to improved plant growth and biomass under Cr and Pb stress, possibly due to their ability to improve nutrient availability, produce phytohormones such as indole-3-acetic acid (IAA), and modulate root architecture, thereby enhancing nutrient and water uptake. Similarly, increased chlorophyll content and improved gas exchange characteristics observed following the application of *P. putida*, *B. amyloliquefaciens*, and *P. fluorescens* may be associated with improved nitrogen availability and cytokinin-mediated protection of chlorophyll pigments under stress conditions, which is in agreement with previous reports on PGPR-mediated enhancement of photosynthetic efficiency^66,67^. Furthermore, the present findings support earlier studies showing that microbial application enhances antioxidant defense systems and reduces oxidative damage under heavy metal stress conditions^1,2,48,68–70^. In the current study, these PGPR may reduce reactive oxygen species (ROS) generation by enhancing antioxidant enzymes, including SOD, POD, CAT, and APX, together with non-enzymatic compounds such as phenolics, flavonoids, ascorbic acid, anthocyanins, and soluble sugars, thereby protecting cellular components from oxidative injury. In addition, *P. putida, B. amyloliquefaciens*, and *P. fluorescens* may influence the AsA–GSH cycle and cellular redox homeostasis under metal stress conditions by modulating antioxidant-related metabolites and stress-responsive defense systems^71–73^. The role of these PGPR in reducing Cr and Pb accumulation is another important mechanism involved in mitigating metal toxicity^74^. Similar findings have also been reported previously, where PGPR reduced heavy metal uptake through immobilization and chelation processes within the rhizosphere^11,75^. Moreover, these PGPR may facilitate the compartmentalization of Cr and Pb into vacuoles and other less metabolically active cellular compartments, thereby minimizing their interference with essential metabolic activities within the cytosol^76,77^. This compartmentalization may be associated with improved cellular fractionation, where toxic metal ions are sequestered into inactive cellular sites, ultimately reducing their toxic effects on critical physiological and biochemical functions^11^. Furthermore, *P. putida*, *B. amyloliquefaciens*, and *P. fluorescens* may also influence proline metabolism, which is associated with osmotic adjustment, stabilization of proteins and cellular membranes, and ROS scavenging under stressed conditions^62,78,79^. The regulation of proline metabolism, together with enhanced antioxidant defense systems and reduced Cr and Pb accumulation, highlights the multifaceted role of these PGPR in improving plant growth, physiological performance, and stress tolerance under Cr and Pb toxicity, ultimately leading to enhanced growth and biomass production in *O. sativa*^48,80^. Among the tested PGPR strains, *B. amyloliquefaciens* exhibited comparatively stronger effects on several morphophysiological and antioxidant attributes under severe Cr and Pb stress conditions. This comparatively improved performance may be associated with its higher capability to produce phytohormones, improve nutrient mobilization, and stimulate antioxidant defense responses under stressed environments, as reported in previous studies^81,82^. However, all tested PGPR strains significantly contributed to stress alleviation and improved physiological performance in *O. sativa*. Although the present study demonstrated significant physiological and biochemical responses associated with PGPR application under Cr and Pb stress, further transcriptomic and molecular investigations are required to fully elucidate the underlying regulatory mechanisms. Nevertheless, the findings of this study highlight the potential application of *P. putida*, *B. amyloliquefaciens*, and *P. fluorescens* as sustainable and eco-friendly biological agents for improving *O. sativa* productivity in metal-contaminated agricultural soils, which is consistent with recent advances in stress-responsive defense regulation and sustainable agricultural approaches^43,83,84,86^. These PGPR-mediated responses may operate through several physiological and biochemical mechanisms, including (1) enhancement of antioxidant and non-antioxidant defense systems, (2) regulation of proline metabolism and AsA–GSH cycle components associated with stress tolerance, and (3) reduction in the translocation and accumulation of Cr and Pb within root and shoot tissues.

## Conclusion

The results of this study reveal the effects of *Pseudomonas putida*, *Bacillus amyloliquefaciens*, and *Pseudomonas fluorescens* application on chromium (Cr) and lead (Pb)-stressed rice (*Oryza sativa* L.). The findings demonstrated that *O. sativa* exhibits adaptive tolerance responses to metal stress, which are associated with activation of antioxidant defense systems. The application of *P. putida*, *B. amyloliquefaciens*, and *P. fluorescens* improved plant growth and biomass, photosynthetic pigment accumulation, gas exchange attributes, sugar contents, AsA–GSH cycle activity, cellular fractionation, and proline metabolism while reducing oxidative stress and metal-induced toxicity in *O. sativa* plants. Furthermore, the application of these PGPR strains enhanced plant tolerance against Cr and Pb stress through improved physiological, biochemical, and stress-responsive defense mechanisms compared with untreated stressed plants. Overall, this study highlights the potential role of PGPR as sustainable biological agents for improving plant performance under metal-stressed conditions. However, further investigations under different crop species and environmental conditions are required to better understand the underlying mechanisms and optimize PGPR application strategies in metal-contaminated soils.

## Data Availability

Data availability: The datasets used and/or analysed during the current study available from the corresponding author on reasonable request..
